# Gabapentin-Induced Cutaneous Leukocytoclastic Vasculitis: A Case Report

**DOI:** 10.7759/cureus.44616

**Published:** 2023-09-03

**Authors:** Ana Órfão, Daniela Madeira, Daniel Maia Duarte, Filipa Galante Pereira, Clara Matos

**Affiliations:** 1 Internal Medicine, Hospital Prof. Doutor Fernando Fonseca, Amadora, PRT; 2 General Medicine, Hospital Prof. Doutor Fernando Fonseca, Amadora, PRT; 3 Anatomical Pathology, Hospital Prof. Doutor Fernando Fonseca, Amadora, PRT

**Keywords:** skin biopsy, hypersensitivity vasculitis, small vessel vasculitis, leukocytoclastic vasculitis, gabapentin

## Abstract

Leukocytoclastic vasculitis (LCV) is a type of small vessel vasculitis, characterized by a perivascular neutrophilic inflammatory infiltrate with fibrinoid necrosis and fragmentation of nuclei (“leukocytoclasia”). Although up to half of the cases of LCV are idiopathic, infections and drugs are the most common secondary triggers for this condition. We present the case of an 88-year-old woman who developed an erythematous maculopapular rash on both thighs three days after starting gabapentin for neuropathic leg pain, without other associated symptoms. Skin biopsy was compatible with cutaneous vasculitis with a leukocytoclastic pattern. The skin lesions resolved within about 10 days after discontinuing gabapentin, supporting the diagnosis. To our knowledge, there are only four published cases of LCV secondary to gabapentin. This case highlights the importance of being alert for diagnosing drug-related cutaneous manifestations, even if the drug is used in our daily practice and vasculitis is not a common side effect, since discontinuing the suspected agent is crucial to resolve skin lesions and to avoid more serious complications.

## Introduction

Cutaneous small vessel vasculitis refers to a small vessel vasculitis confined to the skin, with no systemic involvement or glomerulonephritis [1,2]. Leukocytoclastic vasculitis (LCV) is a type of small vessel vasculitis characterized histopathologically by neutrophilic infiltration into the vessel wall, which causes the disintegration of their nuclei and forms nuclear dust (leukocytoclasia), and deposition of fibrin on the vascular walls, which can result in fibrinoid necrosis [1-3]. Additionally, since the vascular wall and adjacent tissue are damaged, red blood cells may leak out and endothelial cells may become damaged [3]. The LCV pathogenesis involves the presence of circulating immune complexes (mainly of IgG and IgM classes), which activate the complement cascade in small vessel walls [3,4].

Cutaneous LCV frequently presents by a palpable purpura which normally appears on the lower limbs as a result of venous stasis [4-6], although areas subject to gravity, like the back in bedridden patients, can also be involved [3]. Systemic symptoms, such as fever, are absent.

Up to half of LCV cases are idiopathic [4,7,8], and the remaining are usually associated to autoimmune diseases, infections, malignancy and medications [4,5,7]. The most common medications implied are antibiotics (mainly beta-lactams), sulfonamides (namely thiazides), phenytoin, and allopurinol, although many other compounds have been reported [3,5,7,9,10]. Gabapentin is a gamma-aminobutyric acid (GABA) analog commonly used for its anticonvulsant and analgesic effects. Its indications include postherpetic neuralgia, focal onset seizures, restless leg syndrome, neuropathic pain, and fibromyalgia, among others [11]. Despite its structural similarity to the inhibitory neurotransmitter GABA, its mechanism of action is not fully understood [11].

To our knowledge, we present the fifth published case of LCV secondary to gabapentin [5,12-14].

## Case presentation

**Figure 1:**
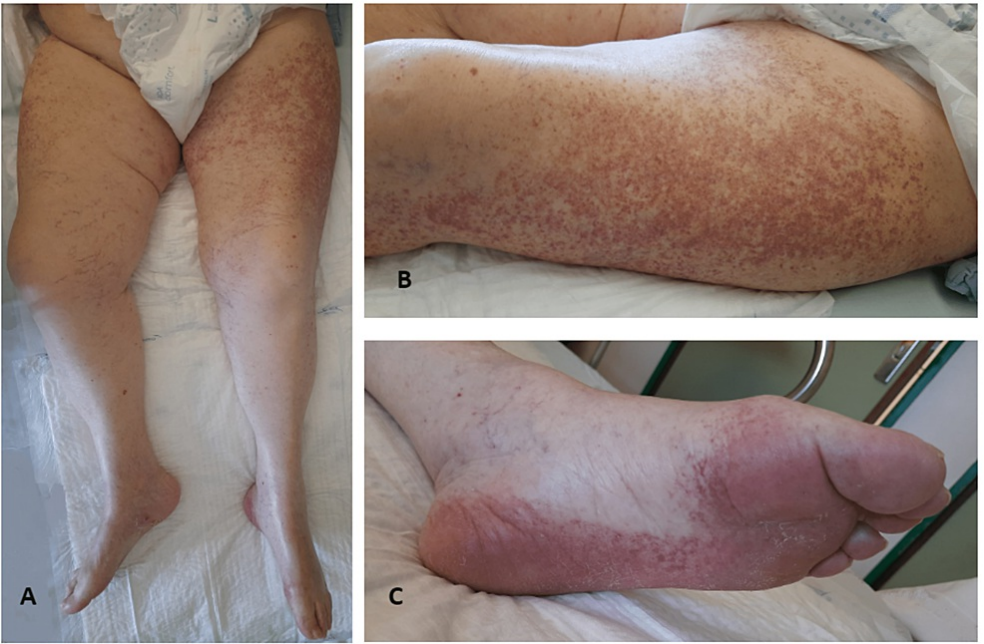
Non-palpable purpura localized in the lower limbs (A), mainly in the left thigh (B) with extension to the foot with plantar involvement (C), suggestive of cutaneous vasculitis.

We present the case of an 88-year-old woman, with a previous history of arterial hypertension and dyslipidemia, who was admitted to the emergency department after recurrent falls in the previous weeks, the last one with traumatic head injury, without loss of consciousness or other associated complaints. On examination, the patient was febrile and was found to have a systolic murmur, without irradiation, bilateral basal rales, and bilateral symmetric oedema of the legs with Godet sign. The initial exams revealed leucocytosis with neutrophilia, elevated C-reactive protein (CRP) of 12,01 mg/dL and elevated N-terminal prohormone of brain natriuretic peptide (NT-proBNP) of 34287 pg/mL (Table 1), and urinalysis showed leukocyturia and nitrituria. The diagnoses of acute pyelonephritis and congestive heart failure were assumed. Urine culture isolated *Escherichia coli*, which was treated with a seven-day course of ceftriaxone. During this time, the patient developed new-onset atrial fibrillation with rapid ventricular response. She was started on furosemide, amiodarone, and bisoprolol, with clinical improvement. Anticoagulation with apixaban was also started to prevent systemic embolism (CHADS-VASc score 5). The patient underwent transthoracic echocardiography, which documented severe aortic stenosis and a reduced left ventricular ejection fraction of 34%. In this context, the diagnosis of symptomatic severe aortic stenosis could justify the recurrent falls due to low brain perfusion, the congestive heart failure, and the atrial fibrillation. She was consequently submitted to percutaneous aortic valve implantation. Afterwards, the patient remained hospitalized undergoing physical therapy and awaiting a place in a rehabilitation unit. On the 47^th^ day of hospitalization, due to complaints of pain in the legs, with reduced tactile and proprioceptive sensitivity, gabapentin 100 mg once a day was started. Three days later, the patient developed a non-palpable, non-blanching, non-tender, non-pruritic erythematous maculopapular rash on her thighs. The rest of her examination was unremarkable. In the next few days, the rash grew in dimensions and progressed to the legs, feet (including soles), and upper limbs (Figure 1). Assuming probable allergic vasculitis secondary to the recently introduced gabapentin, the drug was discontinued.

**Table 1:** Laboratory investigation of the patient. TSH: thyroid stimulating hormone, C4: component 4, C3: component 3, Ig: immunoglobulin, anti-CCP2: anti-cyclic citrullinated peptides 2, Ab: antibody, Sm: Smith, Ag: antigen, anti-P: antiribosomal P protein, c-ANCA: cytoplasmic anti-neutrophil antibody, p-ANCA: perinuclear anti-neutrophil antibody, MPO: anti-myeloperoxidase, anti-PR3: anti-proteinase 3, HCV: hepatitis C virus

Parameter (unit)	Admission	Four days after first skin lesions	Normal Range
Haemoglobin (g/dL)	11.7	11.6	12 - 15
Leucocytes (x 10^9/L)	11,800	6,000	4,000 - 10,000
Eosinophils (x 10^9/L)	0.2	0.4	< 0.6
Platelets (x 10^9/L)	280	283	150 - 410
Sedimentation rate (mm/h)		127	< 20
CRP (mg/dL)	12.01	1.27	< 0.5
NT-proBNP (pg/mL)	34,287	1,010	< 738
TSH (mUI/L)	2.430		0.27 - 4.2
Complement C4 (mg/dL)		26.2	10 - 40
Complement C3 (mg/dL)		106	90 - 180
IgG (mg/dL)		1,333	700 - 1,600
IgA (mg/dL)		529.55	70 - 400
IgM (mg/dL)		62.5	40 - 230
Anti-CCP2		Negative	
ANA		Positive (1:160)	
Anti-SSA (Ro) Ab		Negative	
Anti-SSB (La) Ab		Negative	
Anti-Sm Ab		Negative	
Anti-dsDNA Ab		Negative	
Anti-histones Ab		Negative	
Anti-ribossomal P Protein Ab		Negative	
ANCA			
c - ANCA		Negative	
p - ANCA		Negative	
Anti-MPO Ab		Negative	
Anti-PR3 Ab		Negative	
Anti HIV-1/2 Ab / p24 Ag		Non-reactive	
Hepatitis C - anti-HCV Ab		Non-reactive	
Hepatitis B - HBs Ag		Non-reactive	
Hepatitis B - HBs Ab		Positive	
Hepatitis B - HBc Ab		Reactive	
Cryoglobulins		Negative	

Blood investigations disclosed an increased level of erythrocyte sedimentation rate (ESR) (127 mm/h) and eosinophil count was normal. Investigations done to exclude other causes of the rash included hepatitis B and C, human immunodeficiency virus (HIV), cryoglobulins, anti-ds DNA antibody, and anti-neutrophil cytoplasmic antibody (ANCA), which were negative. Complement (C3, C4) was normal. Antinuclear antibody (ANA) was positive at a titer of 1:160 (linear filamentous cytoplasmic and nucleolar patterns) and immunoglobulin A was increased (575 mg/dL). Urinary sediment revealed rare desquamation epithelial cells, some white blood cells, rare erythrocytes, and some yeasts.

**Figure 2:**
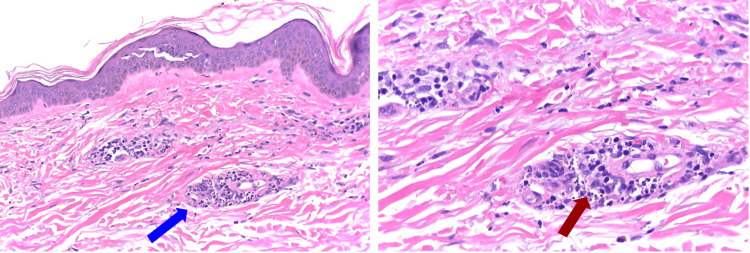
Cutaneous leukocytoclastic vasculitis. The skin fragment shows mild to moderate inflammatory infiltrate of the mixed type in the perivascular stroma (blue arrow), extending through the superficial vascular plexus. The infiltrate consists primarily of neutrophils and some associated lymphocytes, plasma cells, and rare eosinophils. Fragmented neutrophilic nuclei (red arrow), indicative of leucocytoclasis, are present. Additionally, red blood cell extravasation into the interstitium is observed. No thrombi or fibrinoid necrosis were identified. (Hematoxylin & eosin, 200x and 400x, respectively.)

Skin biopsy (Figure 2), performed four days after first skin manifestations, showed mild to moderate inflammatory infiltrate of the mixed type in the perivascular stroma, running through the superficial vascular plexus, consisting of a predominance of neutrophils, plasmocytes, and rare eosinophils, with some nuclear dust being documented. There was disruption of the vessel wall with extravasated erythrocytes into the interstitium and no thrombi or fibrinoid necrosis were identified. Immunoglobulins (IgA, IgG, and IgM) and complement (C1q and C3) in the vessel wall, by direct immunofluorescence, were negative. The pathological diagnosis was cutaneous vasculitis with a leukocytoclastic pattern.

**Figure 3:**
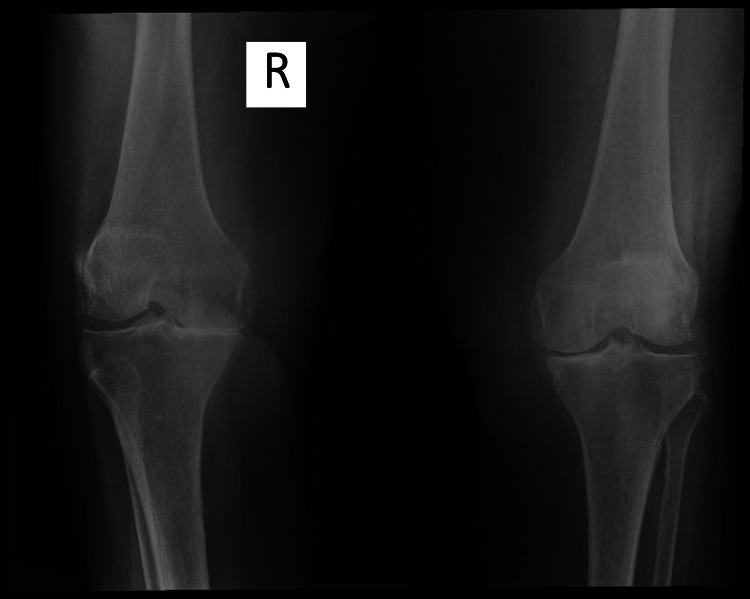
Bilateral knee radiograph showing joint space narrowing in both knees, more accentuated in the medial aspect, and subchondral sclerosis, suggesting gonarthrosis. R: right.

A bilateral knee radiograph was taken to further investigate the pain, showing asymmetric narrowing of the joint space and subchondral sclerosis, suggesting gonarthrosis (Figure 3).

**Figure 4:**
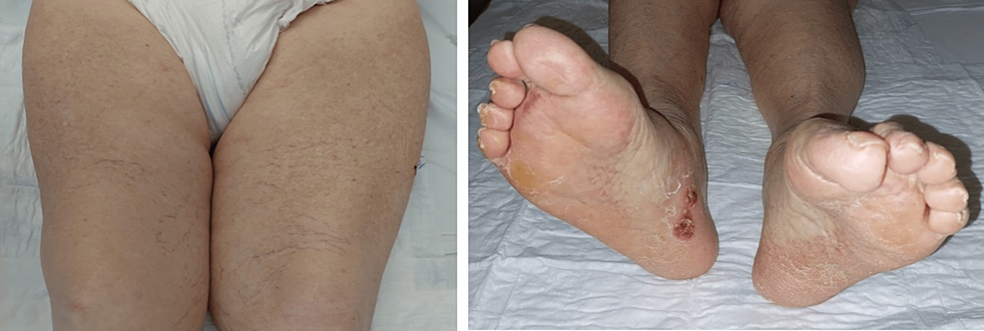
Rash improvement six days after stopping gabapentin.

Other causes of neuropathic pain were excluded (namely diabetes, vitamin B12 deficit, hypothyroidism, or HIV). The pain was controlled with acetaminophen and metamizole, and the sensitive changes resolved spontaneously. With no need for additional treatment, there was a progressive improvement of the rash (Figure 4), and the patient was later discharged to a rehabilitation unit.

Two months after discharge, the patient had a follow-up appointment and the skin lesions were entirely resolved, without further recurrence, and blood tests revealed a decrease in ESR to 70 mm/h.

## Discussion

Cutaneous vasculitis arises as a result of the inflammation affecting dermal blood vessels and clinical manifestations vary based on affected blood vessels (location and size), and the extent of inflammation [6].

Cutaneous LCV usually manifests as papulous or polymorphic purpura and, less often, non-palpable purpura [6]. In the present case, the rash presented as a non-palpable purpura and affected particularly the lower limbs (as shown in Figure 1).

A skin biopsy is crucial to reach a definitive diagnosis [3,4,7,8], as the differential diagnosis can be challenging, especially between other small-vessel vasculitis [4]. In our case, skin biopsy was compatible with leukocytoclastic small vessel vasculitis. Additional laboratory tests excluded systemic immune-mediated disorders and infectious diseases. Given the elevated serum IgA, direct immunofluorescence was performed, which was negative, excluding IgA vasculitis.

In our case, considering the timing correlation between the beginning of gabapentin and the onset of skin lesions, and after excluding other secondary causes (as shown in Table 1), the diagnosis of gabapentin-associated LCV was suspected. When LCV is secondary to a drug or infectious agent, symptoms usually begin seven to 10 days after antigen exposure, the time required to produce enough antibodies to produce antigen-antibody complexes [8]. However, this period may be as short as two to seven days with secondary antigen exposure, which was what happened in our case, suggesting a probable previous exposure to gabapentin, which the patient could not recall.

Apart from gabapentin, there were no other recently introduced medications. Gabapentin is usually a safe and well-tolerated drug with a low incidence of adverse reactions [5]. In opposition to other antiepileptic drugs, skin reactions are rare, and, in adults, the prevalence of rash possibly related to gabapentin varies from 1 to 10% [15].

Besides the temporal association of symptom onset and drug intake, an important factor in suspecting drug-induced cutaneous LCV is the eventual reversibility after discontinuing the drug and the absence of recurrence without further exposure (compare Figures 1, 4) [3].

Considering the criteria proposed by the American College of Rheumatology in 1990 to diagnose hypersensitivity vasculitis [16], our patient fulfilled four out of five criteria: (1) age >16 at disease onset; (2) history of taking a medication at onset that may have been a precipitating factor; (3) the presence of maculopapular rash; and (4) a biopsy demonstrating granulocytes around an arteriole or venule. Furthermore, based on the World Health Organization-Uppsala Monitoring Center (WHO-UMC) causality assessment system [17] and the Naranjo probability scale [18], our case is classified in the 'probable' causality category.

The management of LCV depends on the aetiology and the extent of the disease, the latter reflected by the severity of symptoms, the presence of extracutaneous involvement, or complications. Treatment can range from symptomatic therapy to immunosuppressive medications [3,4,7]. The first step in managing LCV involves treating the underlying cause or discontinuing any identifiable trigger [3,4,7].

In the case of skin-limited disease, the management should focus on symptomatic relief, as most acute episodes are self-limited, resolve in days to weeks without sequelae, and do not recur, even without treatment [3,4,7,8]. General measures include avoiding prolonged standing or walking, leg elevation (if the rash involves dependent areas), use of compression stockings, and avoiding cold exposure [3,4]. In case of pain and pruritus, non-steroidal anti-inflammatory drugs and antihistamines can be useful [4,7].

As in the presented case, in general, cutaneous LCV has a good prognosis, especially if there is an identifiable cause. In drug-induced cutaneous LCV, discontinuing the causative drug is typically resolutive [3,8]. However, approximately 10% of cases experience recurrent or chronic involvement [8]. In the presented case, skin lesions completely resolved within 10 days after discontinuing gabapentin with supportive treatments only, and without recurrence until now.

On the other hand, when cutaneous LCV is severe, refractory, or recurrent, the treatment approach might encompass systemic corticosteroids, potentially combined with adjunctive therapies, like colchicine, dapsone, hydroxychloroquine or immunosuppressive medications, such as mycophenolate or azathioprine [3,4,7].

## Conclusions

LCV is a rare form of small-vessel vasculitis that can be precipitated by commonly used drugs. When suspected, it is crucial to obtain a skin biopsy to confirm the diagnosis.

We describe a rare case of gabapentin-induced cutaneous LCV, highlighting the importance of a high suspicion index for the diagnosis of drug-induced cutaneous reactions, since discontinuing the suspected agent is crucial to resolve skin lesions and to avoid more serious complications.
